# Changes in the Intestine Microbial, Digestive, and Immune-Related Genes of *Litopenaeus vannamei* in Response to Dietary Probiotic *Clostridium butyricum* Supplementation

**DOI:** 10.3389/fmicb.2018.02191

**Published:** 2018-09-19

**Authors:** Yafei Duan, Yun Wang, Hongbiao Dong, Xian Ding, Qingsong Liu, Hua Li, Jiasong Zhang, Dalin Xiong

**Affiliations:** Key Laboratory of South China Sea Fishery Resources Exploitation & Utilization, Ministry of Agriculture and Rural Affairs, Key Laboratory of Fishery Ecology and Environment, Guangdong Province, South China Sea Fisheries Research Institute, Chinese Academy of Fishery Sciences, Guangzhou, China

**Keywords:** *Litopenaeus vannamei*, *Clostridium butyricum*, intestine microbial, metabolism, gene expression

## Abstract

The intestine barrier serves as the front-line defense in shrimp. *Clostridium butyricum* (CB) can produce butyric acid that provides energy for the intestine epithelial cells of the host. However, the effects of dietary CB on the intestine microbiome and the digestion and immunity of the host is not clear. In this study, we therefore investigated the composition and metabolic activity of the intestine microbiome, and digestive and immune-related gene expression in *Litopenaeus vannamei* fed with diets containing different levels of CB: basal diet (control), 2.5 × 10^9^ CFU kg^−1^ diet (CB1), 5.0 × 10^9^ CFU kg^−1^ diet (CB2), and 1.0 × 10^10^ CFU kg^−1^ diet (CB3) for 56 days. Dietary CB altered the composition of the intestine microbiome. Specifically, the dominant bacterial phylum Proteobacteria was enriched in the CB3 group and weakened in the CB1 and CB2 groups. The Bacteroidetes was enriched in the CB1 and CB2 groups and weakened in the CB3 group. The Firmicutes was enriched in all three CB groups. At the genus level, the potential pathogen (*Desulfovibrio* and *Desulfobulbus*) were weakened, and beneficial bacteria (*Bacillus*, *Clostridium*, *Lachmoclostridium*, *Lachnospiraceae*, and *Lactobacillus*) were enriched in response to dietary CB; these might contribute to the expression of the host digestive genes (α-amylase, lipase, trypsin, fatty acid-binding protein, and fatty acid synthase) and immune-related genes (prophenoloxidase, lipopolysaccharide and β-1,3-glucan binding protein, lysozyme, crustin, and superoxide dismutase). Additionally, CB enhanced the bacterial metabolism, especially that of carbohydrates, polymers, amino acids, carboxylic acids, and amines. These results revealed that dietary CB had a beneficial effect on the intestine health of *L. vannamei* by modulating the composition of the intestine microbiome, enhancing the microbial metabolism activity, and promoting the digestion and immunity of the host. The optimal dietary supplementation dosage was found to be 5.0 × 10^9^ CFU kg^−1^ in the diet.

## Introduction

The Pacific white shrimp *Litopenaeus vannamei* is a commercially important shrimp in Southeast Asia (Duan et al., 2017b). In recent years, frequent outbreaks of diseases have caused serious economic losses to shrimp aquaculture (Flegel, 2012; Joshi et al., 2014; Shi et al., 2016), for which effective control measures are still lacking. Antibiotics, vaccines, and chemotherapeutics are all typically used for disease control, but the misuse of these agents has allowed for the emergence of drug-resistant pathogens and can promote animal intestine microbial flora imbalance problems (Sapkota et al., 2008). Therefore, the development of ecofriendly disease preventative approaches will be beneficial to shrimp aquaculture.

The intestine is important for shrimp immunity. It is continuously exposed to foreign substances, including microbes, pathogens, and other toxic substances from food (Li et al., 2007; Rungrassamee et al., 2014). The intestine barrier serves as the front-line immune defense of shrimps and clearly affects shrimp health. The shrimp intestine also harbor a diverse microbial community. The functional activity and stability of the shrimp microbiome is important their health, as it performs many functions related to immunity and pathogen resistance (Duan et al., 2018). Intestine commensal microbes can produce short-chain fatty acids (SCFAs) during their catabolism process (Koh et al., 2016). SCFAs, especially butyric acid, can provide energy for the regeneration and repair of the intestine epithelial cells (Becattini et al., 2016). Additionally, SCFAs can also slightly reduce the intestine pH, promote the growth of probiotic bacteria, and inhibit the growth of pathogenic bacteria (Duan et al., 2017c).

As a Gram-positive butyric acid-producing probiotic, *Clostridium butyricum* is an obligate anaerobic, endospore-forming bacteria and is part of the normal intestine flora for both humans and shrimp (Douglas et al., 1973; Cao et al., 2012; Sumon et al., 2018). CB is very tolerant to low pH and relatively high bile concentrations and temperature environments and has been used as a good feed additive (Zhang et al., 2016). CB can also produce prebiotics, including bacteriocin, lipoteichoic acid, and hydrogen, all of which serve to regulate animal antioxidation and antibacterial functions (Pan, 2006; Gao et al., 2011; Junghare et al., 2012). It was reported that CB and its lipoteichoic acid components could inhibit the growth of pathogens, including *E. coli*, *Salmonella enteritidis*, and *Vibrio parahaemolyticus* (Gao et al., 2011, 2013). In vertebrates, CB could improve the growth, antioxidation, and immune function of broilers (Liao et al., 2015a,b; Zhang et al., 2016). Importantly, heat-killed CB retains interesting immunomodulating properties in *Miichthys miiuy* (Pan et al., 2008). In shrimp, CB-incorporated diets were beneficial for *M. rosenbergii* culture in terms of hindering the growth of *V. harveyi*, and increasing the growth and digestive enzyme activities of shrimp (Sumon et al., 2018). Dietary CB also enhanced the intestine antioxidant capacity and resistance to high temperature stress in *Marsupenaeus japonicus* (Duan et al., 2017a). In our previous studies, we found that the dietary CB promoted growth, increased epithelial cells height and SCFA content, and the enhanced immune function of the intestines of *L. vannamei* against ammonia stress and health in *L. vannamei* (Duan et al., 2017b). It is reasonable to hypothesize that CB can regulate the intestine health of shrimps; however, the effects of CB on the intestine microbial of *L. vannamei* remain unknown.

Digestive enzymes, including α-amylase (Amy), lipase (Lip) and trypsin (Tryp), play very important roles in the digestion of nutrient materials (Hoseinifar et al., 2017). Fatty acid-binding protein (FABP) and fatty acid synthase (FAS) are key enzymes of fatty acid biosynthesis and are involved in the lipid metabolism process (Yang et al., 2011). The antibacterial molecule crustin (Crus) and the prophenoloxidase (proPO) system contribute to the antibacterial capabilities of shrimp (Miandare et al., 2017). LPS and β-1,3-glucan binding protein (LGBP) can recognize LPS and β-1,3-glucan from gram-negative bacteria and is involved in the activation of shrimp immunity (Chen et al., 2016). Lysozyme (LSZ) can destroy peptidoglycan support and cause bacterial splitting under osmotic pressure within bacteria. LSZ thus plays an important role in the shrimp immune defense (Duan et al., 2017b). As an important antioxidant enzyme, superoxide dismutase (SOD) provides the first line of ROS elimination from cells (Zheng et al., 2017). Therefore, these indices can be used to evaluate the effects of dietary *CB* on the digestion and immunity of *L. vannamei*.

In this study, we investigated the effects of dietary CB on intestine health regulation in *L. vannamei*, in terms of the intestine microbial composition, bacterial metabolism activity, and the host digestive and immune-related genes expression. Our results provide information to enhance the understanding the role of CB in the regulation of intestine health of *L. vannamei*.

## Materials and Methods

### Shrimp and Culture Conditions

The experiment was carried out from 2016-07-01 to 2016-09-15. Healthy juvenile *L. vannamei*, with an average weight of 2.36 ± 0.12 g, were collected from a local hatchery and reared in a semi-intensive culture pond at Shenzhen Base, South China Sea Fisheries Research Institute of Chinese Academy of Fishery Sciences (Shenzhen, China). Shrimps were acclimatized in filtered aerated seawater (salinity 30‰, pH 8.2, temperature 28 ± 0.5°C) for 1 week before the beginning of the experiment, and fed daily with a ratio of 5% of body weight using formulated pellet feed (Haida Feed, Jieyang, China), which was approximately 42% crude protein, 7.8% crude lipid, 12.8% ash, and 8% moisture. One-third of the water in each tank was renewed once daily. Water quality parameters including salinity, pH, dissolved oxygen, and temperature were continually measured throughout the experiment using portable multiparameter meter (YSI, United States).

### Diet Preparation

The CB strain was obtained from Zhongke Biotic, Co., Ltd., China, containing endospores with a count of 1 × 10^9^ colony forming units (CFU) g^−1^. The product is the purebred bacterial form of CB and is preserved in spores. It does not have other contaminated bacterial components. The CFU of CB in the product were determined using the plate count method. Add 10 mg bacterial powder to the 1 ml sterilized water, then diluted within a 10-time doubling dilution, and 100 μl of each dilution was plated on three replicate plates of Reinforced Clostridial Medium (Zhu et al., 2013). After 48 h incubation under anaerobic conditions, the CFU were counted according to the standard methods of American Public Health Association [APHA] (1998).

Monoclonal CB strain grown anaerobically in a liquid fermentation tank at 37°C for 48 h, and then the cells were harvested by centrifugation at 5000 *g* for 20 min at 4°C. The bacterial genome was extracted using bacterial DNA extraction kit (Sangon Biotech, Shanghai, China). The identity of the CB strain by 16S rRNA gene amplification was performed with 16S–23S intergenic spacer regions specific primers CB16F and CB23R (**Table 1**). The 50 μL PCRs contained the template DNA 50 ng, DNA polymerase 0.5 U μl^−1^, 2.0 mM MgCl_2_, 0.4 μM primers, 200 μM dNTP and 1X buffer, added ddH_2_O to a total volume of 50 μl. The PCR reaction conditions were 1 cycle of 94°C for 4 min, 35 cycles of 94°C for 1 min, 58°C for 1 min, and 72°C for 1 min, and 72°C for 10 min.

**Table 1:** Primer sequences used in this study^1^.

Primer name	Sequence (5′–3′)	Accession number
CB16F	GGAGAACCTGCGGCTGGAT	Nakanishi et al., 2005
CB23R	AAATCTCCGGATCTCTGGCT	
515F	GTGYCAGCMGCCGCGGTAA	Parada et al., 2016
806R	GGACTACNVGGGTWTCTAAT	Apprill et al., 2015
proPO-F	TACATGCACCAGCAAATTATCG	AY723296
proPO-R	AGTTTGGGGAAGTAGCCGTC	
LGBP-F	CATGTCCAACTTCGCTTTCAGA	AY723297
LGBP-F	ATCACCGCGTGGCATCTT	
Lys-F	GTTCCGATCTGATGTCCGATG	AY170126
Lys-R	AAGCCACCCAGGCAGAATAG	
Crus-F	GAGGGTCAAGCCTACTGCTG	AY486426
Crus-R	ACTTATCGAGGCCAGCACAC	
SOD-F	TGCCACCTCTCAAGTATGATTTC	DQ005531
SOD-R	TCAACCAACTTCTTCGTAGCG	
FABP-F	CGCTAAGCCCGTGCTGGAAGT	KF471026
FABP-R	CTCCTCGCCGAGCTTGATGGT	
FAS-F	GCGTGATAACTGGGTGTCCT	HM595630
FAS-R	ACGTGTGGGTTATGGTGGAT	
Lip-F	ACTGTCTCCTCTGCTCGTC	DQ858927
Lip-R	ATGGTTTCTGGAATAGGTGTTT	
Tryp-F	CGGAGAGCTGCCTTACCAG	X86369
Tryp-R	TCGGGGTTGTTCATGTCCTC	
Amy-F	CTCTGGTAGTGCTGTTGGCT	AH013375
Amy-R	TGTCTTACGTGGGACTGGAAG	
β-Actin-F	GCCCTGTTCCAGCCCTCATT	AF300705
β-Actin-R	ACGGATGTCCACGTCGCACT	

*^1^proPO, prophenoloxidase; LGBP, lipopolysaccharide and β-1, 3-glucan binding protein; Lys, lysozyme; Crus; crustin; SOD, superoxide dismutase; FABP, fatty acid-binding protein; FAS, fatty acid synthase; Lip, lipase; Tryp, trypsin; Amy, α-amylase.*

Four experimental diets were basal diet (control), basal diet supplemented with different levels of CB: 2.5 × 10^9^ CFU kg^−1^ diet (CB1), 5.0 × 10^9^ CFU kg^−1^ diet (CB2), and 1.0 × 10^10^ CFU kg^−1^ diet (CB3). The bacterial cell counts of the diet were examined every day throughout the experiment using the spread de Man, Rogosa & Sharpe (MRS) plate count method to verify the concentration of the probiotic. The CB is well-grown in MRS, such as the colony has neat edges and acid smell; the diameter is 1.5–2.0 mm; the surface is moist, smooth, and opaque. CB was dissolved in sterile purified water and then sprayed homogeneously with formulated feed pellets at the above ration, and the control diet was sprayed with equivalent sterile purified water (Liao et al., 2015a). The diets were kept at room temperature and air dried under ventilation conditions, and used up within a day.

### Experimental Design and Feeding Trial

The experiments were divided into four groups (control, CB1, CB2, and CB3), and each group included three replicate 500 L fiberglass tanks. There were 30 shrimps per tank. Each tank was covered with a plastic mesh lid to prevent the shrimps from jumping out of the tank. The water was continuously aerated with two air stones in each tank. Before starting the experiment, the health status of the shrimps was examined for signs of infection. The shrimps were fed with these four diets with a ratio of 5% of body weight. The feeds were given three times per day (07:00, 12:00, and 18:00). The light regime was set at a fixed 14 h light and 10 h dark. During the feeding trial, shrimps were fed to nearly satiation, and the uneaten feed particles after feeding 1 h were collected, dried and weighed for the correction of feed intake. The feeding trial lasted for 56 days. At the end of the experiment (56 days), the whole intestines of shrimps from each tank were randomly sampled, snap frozen in liquid nitrogen and stored at −80°C for intestine microbial composition and gene expression analysis. Additionally, the whole intestines from three shrimps from each tank were randomly sampled and stored at 4°C for analysis of the intestine microbial metabolism activity.

### Intestine Microbiome Composition Analysis

Intestine microbial DNA was extracted using a PowerSoil^TM^ DNA Isolation Kit (Mo Bio Laboratories, Inc., Carlsbad, CA, United States) according to the manufacturer’s protocol and analyzed in 1.0% agarose electrophoresis. The purity was quantitated at 260 nm, and all OD_260_/OD_280_ were between 1.8 and 2.0. The amplification of the V4 region of the bacterial 16S rRNA gene was obtained using the barcoded fusion primers 515F (Parada et al., 2016) and 806R (Apprill et al., 2015) (**Table 1**). The 20 μl PCR reactions contained the template DNA 10 ng, 5× FastPfu buffer 4 μl, dNTPs (2.5 mM) 2 μl, primer 515F (5 μM) 0.8 μl, primer 806R (5 μM) 0.8 μl, FastPfu polymerase 0.4 μl, added ddH_2_O to the total volume 20 μl. The PCR reaction conditions were 1 cycle of 95°C for 5 min, 27 cycles of 95°C for 30 s, 55°C for 30 s, and 72°C for 45 s, and 72°C for 10 min. The PCR fragments were subjected to electrophoresis on 1.5% agarose gels to determine length differences, and the target band was purified by a PCR purification kit (Qiagen). The amplicons were pooled in equimolar concentrations and sequenced with an Illumina HiSeq platform.

The raw sequences were processed using the BIPES pipeline. Chimeric sequences were determined by UCHIME (Edgar et al., 2011). The operational taxonomy units (OTUs) were defined with a threshold of 97% identity by UPARSE (Edgar, 2013). Taxonomies were assigned with uclust for each OUT, and alpha and beta diversity analyses were determined for each library using QIIME. The heatmap was constructed by using the heatmap 2 function of the R g-plots package based on the top 100 genera of the samples.

### Intestine Microbiome Metabolism Analysis

The metabolic activity of intestine microbes were analyzed using Biolog^TM^ EcoPlates (Hayward, CA, United States). The Biolog EcoPlates contained 31 carbon sources that be classified into six types, namely, polymers, carbohydrates, carboxylic acids, amino acids, amines, and phenolic compounds (Choi and Dobbs, 1999). There were three replicate sets of carbon substrates and a control. Briefly, intestines from three shrimps from each tank were homogenized by adding sterile 0.9% saline solution to prepare 10% (*w*:*v*) homogenates. Homogenates were centrifuged at 3500 rpm for 10 min at 4°C, and then the suspension was filtered with a cellulose nitrate membrane filters (50 μm pore size). The clear supernatants were diluted 100-fold, and 150 μl was used to inoculate each of the 96 microtiter wells of the Biolog plate. The inoculated plates were incubated at 30°C, and microbial development was followed by reading the optical density (OD) at 590 nm for 0, 24, 48, 72, 96, 120, 144, and 168 h using a Biolog Microstation^TM^ reader (Biolog, Inc., Hayward, CA, United States). Assays were all run in three replicate samples.

The average well-color development (AWCD) of Biolog^TM^ EcoPlates has been used as an index for aerobic metabolism of cultivable bacteria under investigation. The relative utilization efficiency of the 31 carbon sources was evaluated after incubated with the Biolog^TM^ EcoPlates 72 h. AWCD was calculated with the following formula:

$$\mathrm{AWCD}=\sum (C_{i}-R)/n$$

where *C*_i_ is the color production within each well, *R* is the absorption value in the control well, and *n* is the substrate number (31 in EcoPlates).

### Gene Expression Analysis

Total RNA was extracted from intestine without feces of three shrimps in each tank using TRIzol Reagent (Invitrogen, United States) following the manufacturer’s protocol. Contaminant DNA was removed from RNA samples using RQ1 RNase-Free DNase (Promega, United States), and the RNA samples were analyzed in 1.0% agarose electrophoresis and quantitated at 260 nm, all OD_260_/OD_280_ were between 1.8 and 2.0. Total RNA (8 μg) was reverse transcribed to the first-strand cDNA using M-MLV reverse transcriptase (Promega, United States) following the manufacturer’s instruction.

Real time quantitative RT-qPCR was performed using the SYBR^®^ Premix Ex Taq^TM^ II Kit (TaKaRa, Japan) with an ABI PRISM 7500 Sequence Detection System (Applied Biosystems, United States) to investigate the expression of digestive and immune related genes, including *Amy*, *Lip*, *Tryp*, *FABP*, *FAS*, *proPO*, *LGBP*, *Lys*, *Crus*, and *SOD*. The β-*actin* gene of *L. vannamei* was used as an internal control to verify the successful reverse transcription and to calibrate the cDNA template. The qPCR specific primers were designed based on the open reading frame (ORF) of the target genes using Primer Premier 5.0 software (**Table 1**), and the efficiency was evaluated with amplification plot and melt curve. The RT-qPCR was carried out in a total volume of 20 μl, containing 10 μl SYBR^®^ Premix Ex Taq^TM^ II (2×) (TaKaRa), 2 μl of the 1:5 diluted cDNA, 0.8 μl each of 10 μmol/L forward and reverse primers (or β-actin-F and β-actin-R to amplify the β-*actin* gene) (**Table 1**), 0.4 μl ROX Reference Dye II (50×) and 6 μl DEPC-treated water. The PCR program was 95°C for 30 s, then 40 cycles of 95°C for 5 s and 60°C for 34 s, followed by 1 cycle of 95°C for 15 s, 60°C for 1 min and 95°C for 15 s. DEPC-treated water for the replacement of template was used as negative control. RT-qPCR data from three replicate samples were analyzed with the ABI 7300 system SDS Software (Applied Biosystems, United States), for estimating transcript copy numbers for each sample. The relative expression level of gene was expressed as the fold-change in expression, relative to the β-*actin* gene, calculated by the 2^−ΔΔ*CT*^ comparative C_T_ method.

### Statistical Analysis

The value of each variable was expressed as the mean ± SE. Statistical analysis was performed using SPSS software (Ver 17.0). Statistical significance was determined using one-way ANOVA and *post hoc* Duncan multiple range tests. Significance was set at *P* < 0.05. All data were tested for normality, homogeneity and independence before ANOVA.

## Results

### Intestine Microbial Richness and Diversity

**FIGURE 1:**
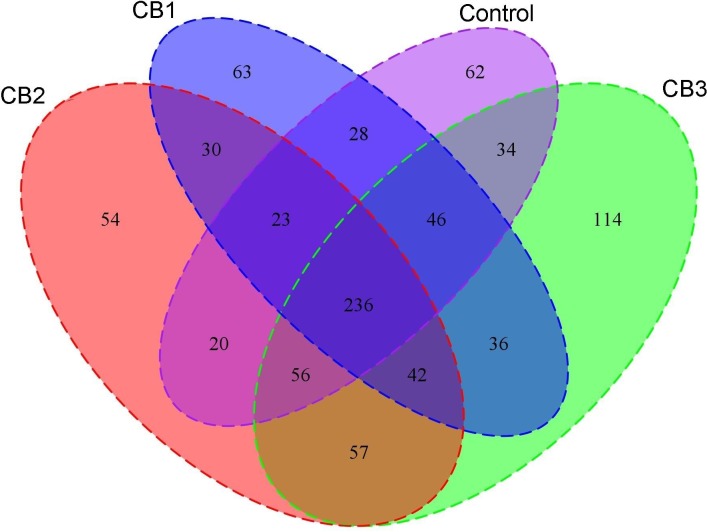
Venn diagrams analysis of intestine microbial with OTUs of the four groups.

A total of 38,567 sequences were obtained from the intestine microbiome of *L. vannamei* using 16S rRNA gene V4 region Illumina sequencing, with an average of 37,577 sequences per sample obtained after optimization and quality control; the percent was 97.43%. The average sequence length was 256 nt. After rarefaction curve analysis, the observed species per sample was sufficient (**Supplementary Figure S1**). A total of 236 OTUs were shared by the four groups with Venn diagram analysis, and the number of unique OTUs in the CB3 group was the highest (**Figure 1**). The alpha diversity analysis showed that the bacterial richness index including ACE and Shannon were increased in the CB2 and CB3 groups; the bacterial diversity indexed by Shannon was increased in the CB1 and CB2 groups and decreased in the CB3 group. However, there were no significant differences. Good’s estimated sample coverage (ESC) in the four groups was 96.42–97.82% (**Table 2**).

**Table 2:** Diversity indices of intestine microbial used in this study^1^.

Group	Observed species	Chao1	ACE	Shannon	ECS (%)
Control	299 ± 22^b^	343 ± 30^a^	299 ± 25^a^	4.04 ± 0.19^a^	97.79 ± 1.44^a^
CB1	236 ± 17^a^	337 ± 119^a^	305 ± 63^a^	4.39 ± 0.40^a^	97.56 ± 2.21^a^
CB2	359 ± 33^c^	397 ± 54^a^	360 ± 51^a^	4.57 ± 0.18^a^	97.82 ± 2.28^a^
CB3	341 ± 7^c^	372 ± 15^a^	342 ± 34^a^	3.81 ± 0.36^a^	96.42 ± 1.76^a^

*^1^ACE, abundance-based coverage estimator; ECS, Good’s estimated sample coverage. Vertical bars represented the mean ± SE (N = 3). Data indicated with different letters were significantly different (P < 0.05) among treatments.*

### Changes in the Intestine Bacterial Composition

**FIGURE 2:**
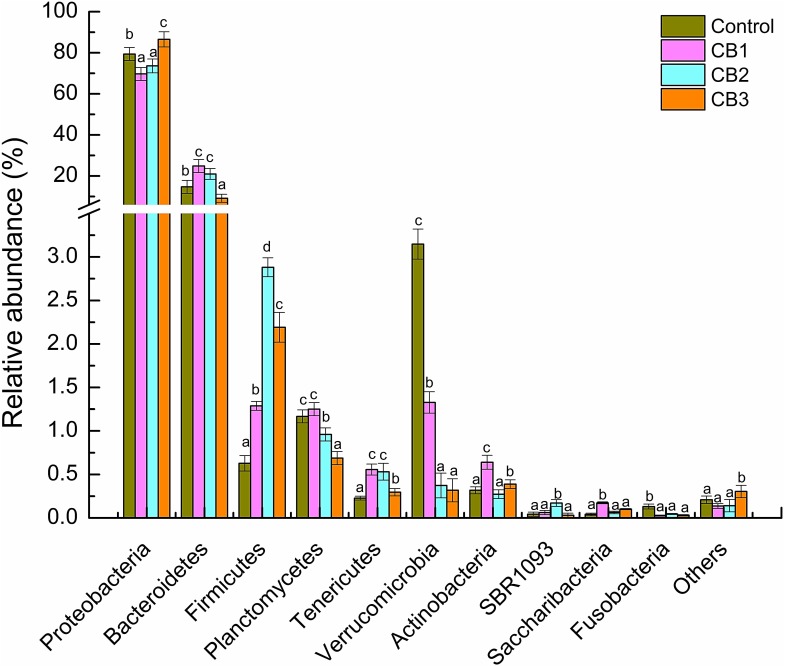
Relative abundance of intestine bacterial of *Litopenaeus vannamei* at the phylum level. Vertical bars represented the mean ± SE (*N* = 3). Data marked with different letters were significantly different (*P* < 0.05) among groups.

A total of 31 different bacterial phylum were identified. Proteobacteria, Bacteroidetes, and Firmicutes were three of the dominant phylum in the four clustered groups. The relative abundance of Proteobacteria was enriched in the CB3 group, and weakened in the CB1 and CB2 groups. The relative abundance of Bacteroidetes was enriched in the CB1 and CB2 groups and weakened in the CB3 group. Firmicutes was abundant in the three CB treatment groups (**Figure 2**).

**FIGURE 3:**
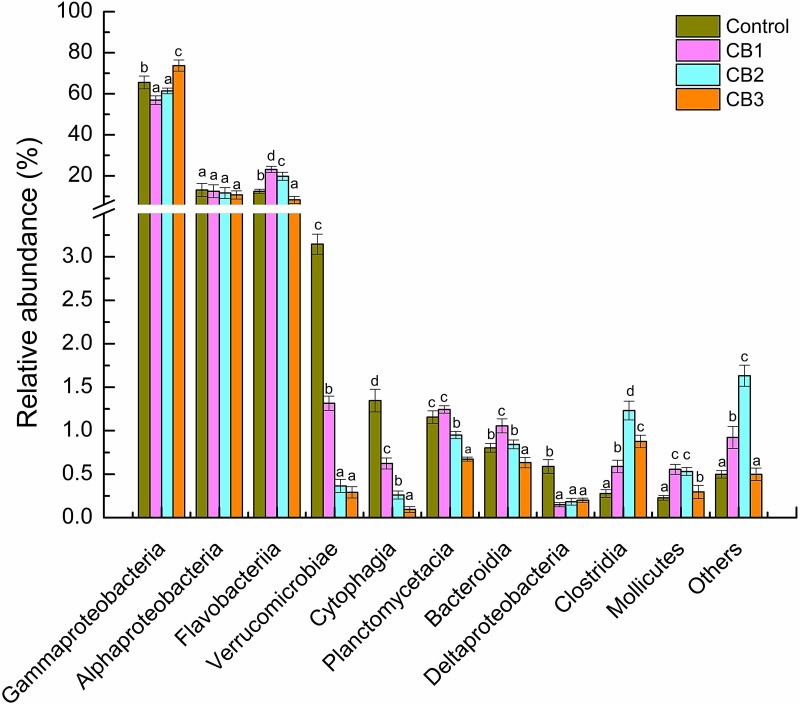
Relative abundance of intestine bacterial of *L. vannamei* at class level. Vertical bars represented the mean ± SE (*N* = 3). Data marked with different letters were significantly different (*P* < 0.05) among groups.

**FIGURE 4:**
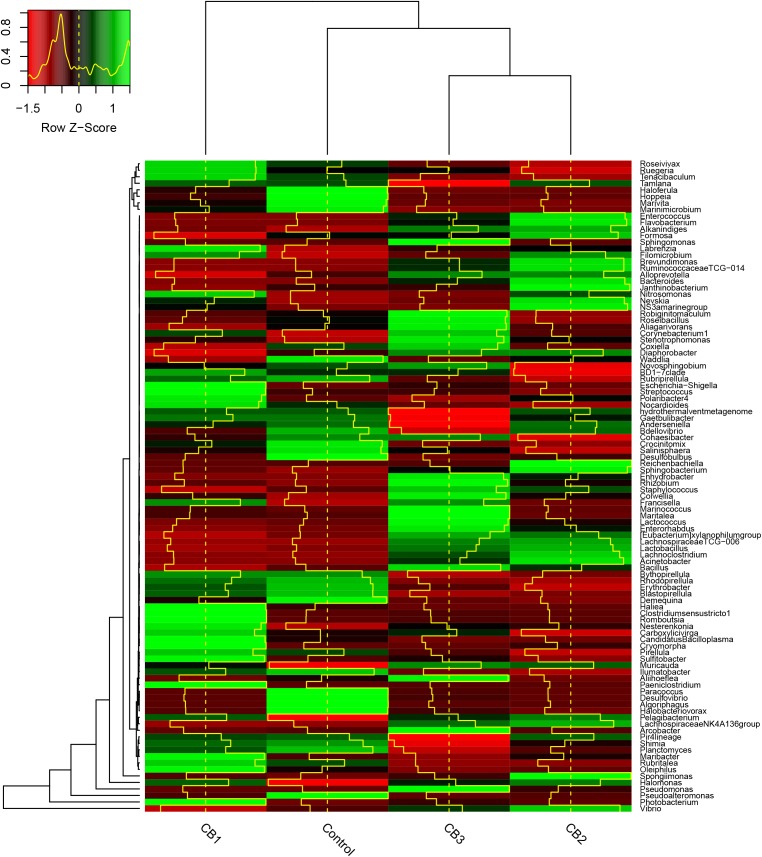
Heatmap of intestine microbial abundance of *L. vannamei* at the genus level. The OTUs were organized per their phylogenetic positions, the taxa of OTUs are shown on the right. The Z-Score indicates the relative abundance of species of each row after standardization in heatmap. The height of the yellow line indicates the Z-score in the color key.

At the class level, Gammaproteobacteria, Alphaproteobacteria, and Flavobacteriia were the primary intestine bacteria in all of the groups assessed. The relative abundance of Gammaproteobacteria was enriched in the CB3 group but weakened in the CB1 and CB2 groups. The relative abundance of Flavobacteriia was enriched in the CB1 and CB2 groups and weakened in the CB3 group. The relative abundance of Alphaproteobacteria was statistically the same in the three CB groups (**Figure 3**). At the genus level, the beneficial bacteria *Clostridium* was enriched in the CB1 group; *Lachmoclostridium*, *Lachnospiraceae*, and *Lactobacillus* were enriched in the CB2 and CB3 groups; and *Bacillus* and *Lactococcus* were enriched in the CB3 groups. Some potential pathogen, such as *Desulfovibrio* and *Desulfobulbus* were absent in the three CB treatment groups (**Figure 4**).

**FIGURE 5:**
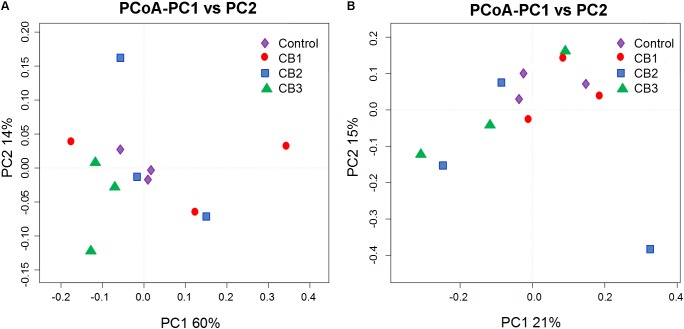
Principal coordinates analysis (PCoA) analysis of the intestine microbial communities in different samples. **(A)** PCoA plots based on weighted UniFrac metrics. **(B)** PCoA plots based on unweighted UniFrac metric.

Principal coordinates analysis (PCoA) of weighted and unweighted UniFrac distances was further constructed to confirm that the intestine bacterial in the control group and the three CB groups were separated (**Figure 5**). The analysis of similarity by ANOSIM showed that Control-CB1-CB3 and Control-CB2 were well-separated groups, CB2-CB1 and CB2-CB3 were overlapping groups, but there were no significant differences (**Table 3**).

**Table 3:** Similarity values (*R*-ANOSIM) between groups of the intestine microbial (*N* = 3)^1^.

	*R*-value	*P*-value
CB1-Control	0.4074	0.1
CB3-Control	0.7037	0.1
CB3-CB1	0.2963	0.1
CB2-Control	0.4074	0.1
CB2-CB1	−0.1481	0.8
CB2-CB3	0	0.5

*^1^R-ANOSIM values between −1 and 1. R > 0 represents well-separated groups. R < 0 represents separated, but overlapping groups. The resulting P-value gives the significance of similarity, and significance was set at P < 0.05.*

### Intestine Bacterial Metabolism Activities

**FIGURE 6:**
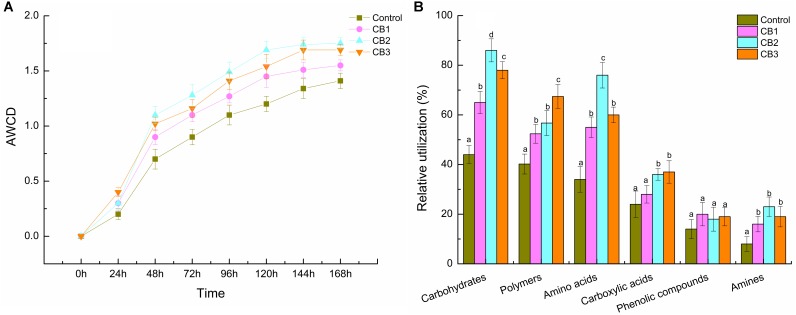
Aerobic metabolism of cultivable microbial in intestine of *L. vannamei* fed the control diet and three CB-containing diets for 56 days. **(A)** AWCD activity; **(B)** relative utilization of six groups of carbon source. Vertical bars represented the mean ± SE (*N* = 3). Data marked with different letters were significantly different (*P* < 0.05) among groups.

The aerobic metabolism of the intestine cultivable bacterial was evaluated using AWCD of Biolog^TM^ EcoPlates. Compared with the control, the AWCD values were increased in the three CB groups, and the highest was in the CB2 group. The AWCD values in the CB1, CB2, and CB3 groups were 1.10-, 1.24-, and 1.20-fold of the control group after cultivation for 168 h respectively (**Figure 6A**). The relative utilization of carbon sources including carbohydrates, amino acids, polymers, and amines were increased in the three CB groups, whereas phenolic compounds were no changes. Carbohydrates, amino acids, and polymers were the highest carbon sources, the relative utilization of which was the highest in the CB2, CB3, and CB2 groups, respectively. The relative utilization of carboxylic acids was statistically the same in the CB2 and CB3 groups. Amines were statistically the same in the three CB groups (**Figure 6B**).

### Expression Levels of Intestine Digestive and Immune Related Genes

**FIGURE 7:**
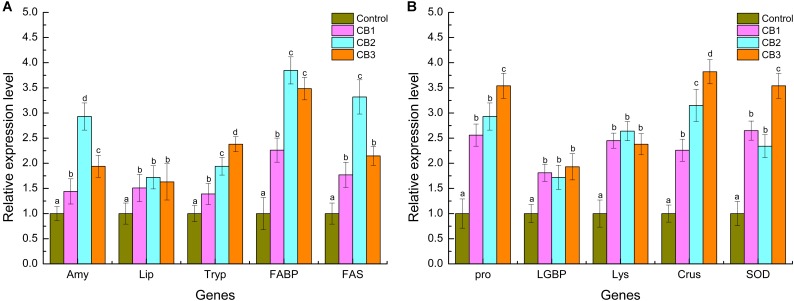
Digestive and immune-related genes expression level in intestine of *L. vannamei* fed the control diet and three CB-containing diets for 56 days. **(A)** Digestive genes; **(B)** immune genes. The reference gene is β-*actin*. Vertical bars represented the mean ± SE (*N* = 3). Data marked with different letters were significantly different (*P* < 0.05) among groups.

Compared with the control, the relative expression levels of digestive related genes including *Amy*, *Lip*, *Tryp*, *FABP*, and *FAS* were increased in the three CB groups. The expression levels of the *Amy*, *FABP*, and *FAS* genes were the highest in the CB2 group, while the expression level of the *Lip* gene was statistically the same in the three CB groups. The expression level of the *Tryp* gene was the highest in the CB3 group (**Figure 7A**).

The relative expression levels of immune-related genes, including *proPO*, *LGBP*, *Lys*, *Crus* and *SOD*, were also increased in the three CB groups. The expression levels of the *proPO*, *Crus*, and *SOD* genes were the highest in the CB3 group, but the expression level of the *LGBP* and *Lys* gene was statistically the same in the three CB groups (**Figure 7B**).

## Discussion

Animal intestines possess a large surface area that provides a barrier to inflammation and pathogen infection. The intestine barrier of animals is associated with structural integrity, immune proteins, and a stable microbiome (Duan et al., 2018). The intestine microbiome contains various opportunistic pathogens that do not cause disease in a healthy host. If the host’s resistance is lowered or the intestine microbiome is in imbalance, the opportunistic pathogens are capable of causing disease. It is thought that CB can regulate the intestine micro-ecological balance of animals (Nakanishi et al., 2003). In this study, 16S rRNA gene illumina sequencing and Biolog^TM^ EcoPlates were used to study intestine microbial composition and metabolism activity in *L. vannamei* fed different does of CB. Our results showed that dietary CB altered the intestine microbial composition of the shrimp, improving the aerobic metabolism of cultivable bacterial, particularly carbon source metabolism, including carbohydrates, amino acids, and polymers.

Following 56 days of supplementation with CB, the dominant intestine bacterial species of the shrimp were *Proteobacteria* and *Bacteroidetes*, which are normally dominant in the intestines of shrimp at all growth stages (Zhang et al., 2014; Huang et al., 2016). Additionally, the Firmicutes abundance was dominant in the three CB groups. Firmicutes bacteria provide a good index regarding the state of the intestine. For example, *Bacillus*, *Lactobacillus*, and *Lactococcus* spp. can prevent the production of inflammatory cytokines and pathogen-induced intestine function disruption (Harzevili et al., 1998; Li et al., 2009; Zheng et al., 2017). *Lachnospiraceae* can participate in carbohydrate fermentation into SCFAs and gasses (CO_2_ and H_2_) in the human intestine (Duncan et al., 2007). In this study, the beneficial bacteria of Firmicutes, such as *Bacillus*, *Clostridium*, *Lachmoclostridium*, *Lachnospiraceae*, and *Lactobacillus*, were abundant in the CB group. Therefore, the results of this study revealed that dietary CB stimulated the growth of beneficial bacteria in the intestines of *L. vannamei*, and its beneficial effects depended on the addition of the dose.

Several studies showed that inactivated or dead probiotics could also stimulate the immunity of aquatic animals (Irianto and Austin, 2003; Salinas et al., 2006; Taoka et al., 2006). Pan et al. (2008) reported that heat-killed *CB* retain interesting immunomodulating properties in *Miichthys miiuy*. It is strange that *Clostridium* is only abundant in the CB1 group, as shown by heatmap analysis, but we could not confirm that the *Clostridium* detected by sequencing was the one added. As shrimp have a short intestine, considering the supply of high numbers of spores, we speculated that CB might not colonize the intestine, only having beneficial effects in the intestine tissues. We will study whether dietary CB can colonize in the intestine of shrimps using fluorescent mark method in the future.

Some opportunistic pathogen genera were absent in response to dietary CB, including *Desulfovibrio* and *Desulfobulbus*. *Desulfovibrio* and *Desulfobulbus* are major sulfate-reducing bacteria (SRB) that are ubiquitous and present in animal and human intestines (Ichiishi et al., 2010). SRB can generate large quantities of hydrogen sulfide (H_2_S) to damage intestine epithelial cells (Gibson et al., 1988; Zhang et al., 2015). These results revealed that CB might decrease the risk of opportunistic pathogens to invade the host immunity. Evidence indicates that *Vibrio* is normally dominant in the intestines of shrimp (Liu et al., 2011; Rungrassamee et al., 2014; Zheng et al., 2016). Although *Vibrio* spp. are potential pathogens, they may not cause shrimp in a healthy host (Richards et al., 2015). *Halobacteriovorax* is also called *Bdellovibrio*, which can act against the growth of *Vibrio* spp. (Richards et al., 2012; Williams et al., 2016). In this study, *Halobacteriovorax* was dominant in the control group, while *Vibrio* was absent. This phenomenon should be further investigated. The bacterial community in the treatments of CB varied from each other, which might be caused by other factors in addition to CB, including water quality and individual variation.

Probiotics can secret a variety of digestive enzymes and improve the digestive function of shrimps (Ziaei-Nejad et al., 2006; Zokaeifar et al., 2012; Miandare et al., 2016). In this study, the relative expression levels of digestive enzyme genes, including *Amy*, *Lip*, and *Tryp* were increased in response to dietary CB, which revealed that CB could enhance the intestine digestive capacity of the host. Fatty acid biosynthesis is essential for the maintenance of cellular homeostasis. Fatty acids produced by cells of host or bacteria are used as an energy source/reserve (Laplante and Sabatini, 2009). Dietary CB could increase the intestine SCFAs contents of *L. vannamei* (Duan et al., 2017b). In this study, the relative expression levels of the *FABP* and *FAS* genes were increased in intestine of *L. vannamei*. Hence, forecasts CB contributed the fatty acid biosynthesis of shrimp, particularly SCFAs.

Probiotics can act as non-specific immune factors and have a positive effect on the host immune system through flagellin, LPS, peptidoglycan and the secretion of cytokines recognized by intestine mucosal cell surface receptor (Kemgang et al., 2014). For example, *B. subtilis* could up-regulate the immune genes expression of *L. vannamei* including *proPO*, peroxinectin (PE), *LGBP* and serine protein (SP) (Zokaeifar et al., 2012). The oral administration of *L. planetarium* also induced the immune responses and gene expression of *L. vannamei* (Chiu et al., 2007). In this study, the relative expression levels of immune genes *propO*, *LGBP*, *Lys*, *Crus*, and *SOD* were increased in the intestines of *L. vannamei*. Additionally, SCFAs can penetrate the pathogenic bacterial cell wall, release its protons (H^+^), and disorder the bacterial cell metabolism, thus cause the bacterial lower cell growth and even cell death (De Schryver et al., 2010). These mechanisms revealed that CB not only could improve the host immunity but could also inhibit the pathogenic bacteria by increasing the SCFA contents in the intestines of shrimp. However, higher concentrations of dietary CB did not produce better effects. Considering economic practicality, CB is a good probiotic and that its optimum dosage is 5.0 × 10^9^ CFU kg^−1^ diet for shrimp intestine health regulation under the present study conditions.

## Conclusion

This study demonstrated that the dietary supplementation of CB modulated the composition and metabolic activity of the intestine microbiome in *L. vannamei*, while some species of beneficial bacteria were abundant, opportunistic pathogens were absent. In addition, CB increased the intestine digestive and immune-related gene expression. These results suggest that CB may benefit intestine health regulation in *L. vannamei*, and is candidate for use as a shrimp feed additive; the optimal dietary supplementation dosage is 5.0 × 10^9^ CFU kg^−1^ diet. Further studies will therefore focus on the effects of CB on the relevance of the intestine microbiome with the host immune system in shrimp.

## Ethics Statement

The collection and handling of the animals in this study was approved by the Animal Care and Use Committee at the Chinese Academy of Fishery Sciences, and all experimental animal protocols were carried out in accordance with national and institutional guidelines for the care and use of laboratory animals at the Chinese Academy of Fishery Sciences.

## Author Contributions

YD conceived, designed, and wrote the manuscript. YD and YW performed the experiments. JZ assisted in the experimental design and preparation of the manuscript. XD, HL, QL, and DX helped to collect the samples and analyze the data. All authors approved the final manuscript.

## Conflict of Interest Statement

The authors declare that the research was conducted in the absence of any commercial or financial relationships that could be construed as a potential conflict of interest.

## Supplementary Material

FIGURE S1 Rarefaction curves analysis of intestine microbial with OTUs of the four groups.
